# Lamin A/C Is a Risk Biomarker in Colorectal Cancer

**DOI:** 10.1371/journal.pone.0002988

**Published:** 2008-08-20

**Authors:** Naomi D. Willis, Thomas R. Cox, Syed F. Rahman-Casañs, Kim Smits, Stefan A. Przyborski, Piet van den Brandt, Manon van Engeland, Matty Weijenberg, Robert G. Wilson, Adriaan de Bruïne, Christopher J. Hutchison

**Affiliations:** 1 School of Biological and Biomedical Sciences, Durham University, Durham, United Kingdom; 2 James Cook University Hospital, Middlesbrough, United Kingdom; 3 Department of Epidemiology, University of Maastricht, Maastricht, The Netherlands; 4 Department of Pathology, University of Maastricht, Maastricht, The Netherlands; University of Edinburgh, United Kingdom

## Abstract

**Background:**

A-type lamins are type V intermediate filament proteins encoded by the gene *LMNA*. Mutations in *LMNA* give rise to diverse degenerative diseases related to premature ageing. A-type lamins also influence the activity of the Retinoblastoma protein (pRb) and oncogenes such a β-catenin. Consequently, it has been speculated that expression of A-type lamins may also influence tumour progression.

**Methodology/Principal Findings:**

An archive of colorectal cancer (CRC) and normal colon tissue was screened for expression of A-type lamins. We used the Cox proportional hazard ratio (HR) method to investigate patient survival. Using CRC cell lines we investigated the effects of lamin A expression on other genes by RT-PCR; on cell growth by FACS analysis; and on invasiveness by cell migration assays and siRNA knockdown of targeted genes. We found that lamin A is expressed in colonic stem cells and that patients with A-type lamin-expressing tumours have significantly worse prognosis than patients with A-type lamin negative tumours (HR = 1.85, *p* = 0.005). To understand this finding, we established a model system based upon expression of GFP-lamin A in CRC cells. We found that expression of GFP-lamin A in these cells did not affect cell proliferation but did promote greatly increased cell motility and invasiveness. The reason for this increased invasiveness was that expression of lamin A promoted up-regulation of the actin bundling protein T-plastin, leading to down regulation of the cell adhesion molecule E-cadherin.

**Conclusions:**

Expression of A-type lamins increases the risk of death from CRC because its presence gives rise to increased invasiveness and potentially a more stem cell-like phenotype. This report directly links A-type lamin expression to tumour progression and raises the profile of *LMNA* from one implicated in multiple but rare genetic conditions to a gene involved in one of the commonest diseases in the Western World.

## Introduction

Lamins A and C are type V intermediate filament proteins that form part of a filamentous network termed the nuclear lamina lining the inner nuclear membrane (INM) [1]. A-type lamins are alternatively spliced products of the *LMNA* gene, which has been mapped to chromosome 1q21.3 [2]. Mutations in this gene are the underlying cause of twelve different genetic diseases that are collectively termed laminopathies [3]. Laminopathies are all degenerative diseases that mainly affect tissues of mesenchymal origin [3]. Possible mechanisms underlying laminopathies have been intensively investigated over the past seven years and this has led to the conclusion that A-type lamins contribute to cell survival in two distinct ways. Firstly, A-type lamins interact with important cytoskeletal linker proteins termed nesprins, via SUN domain proteins, connecting the INM to the outer nuclear membrane (ONM) via the lumen [4], [5]. The nesprins in turn anchor elements of the cytoskeleton to the ONM [6]–[9], thereby hardwiring the cytoskeleton to the nuclear lamina and providing a device for transducing mechanical stress sensing from the plasma membrane to the nucleus [10], [11]. Secondly, A-type lamins interact with a number of binding partners within the nucleus, which in turn interact with and influence the activity of important growth regulators. Of the proteins that A-type lamins interact with, the best characterised are the so-called LEM domain proteins [12], including the integral membrane proteins emerin [13], [14] and MAN1 [15], as well as the nucleoskeleton protein LAP2α [16]. A complex of A-type lamins and emerin has recently been reported to regulate the nuclear accumulation of active β-catenin and loss of emerin function leads to unregulated β-catenin signalling and auto-stimulatory growth in fibroblasts [17]. Similarly, a complex of MAN1 and A-type lamins has been shown to interact with the receptor regulated SMAD (rSMAD) and to antagonise TGF-β signalling by inhibiting rSMAD at the INM [18], [19]. Finally, a complex of LAP2α and A-type lamins binds to and tethers unphosphorylated forms of the growth suppressor pRb in the nucleus [20]. LAP2α and A-type lamins both participate in Rb dependent E2F repression [21] and loss of LAP2α or A-type lamins in fibroblasts results in accelerated S-phase entry, through loss of pRb activity [21], [22].

Given the importance of A-type lamins and their binding partners to the regulation of growth pathways, it has been speculated that these lamins might be linked to tumour progression [23]. Previous studies have reported differential expression of A-type lamins in tumour tissues and have linked the absence of A-type lamins to increased proliferation in the tumour. However, they have failed to link changes in expression to patient prognosis or directly to tumour progression [24]. We therefore decided to investigate how expression of A-type lamins might influence both the progression and outcomes of a common tumour. To do this we screened a very large archive of CRC tissue linked to an extensive patient database [25]. Unexpectedly, we found that expression of A-type lamins within a tumour was a highly significant risk indicator of tumour related mortality. In downstream investigations, we found that expression of lamin A in CRC cell lines promoted invasiveness via up-regulated expression of the actin-bundling protein T-plastin, which in turn gives rise to down-regulated expression of the cell adhesion molecule E-cadherin. We conclude that expression of A-type lamins in CRC promotes tumour invasiveness through reorganisation of the actin cytoskeleton.

## Results

### Lamin A is an adult stem cell biomarker in colonic crypts

**Figure 1:**
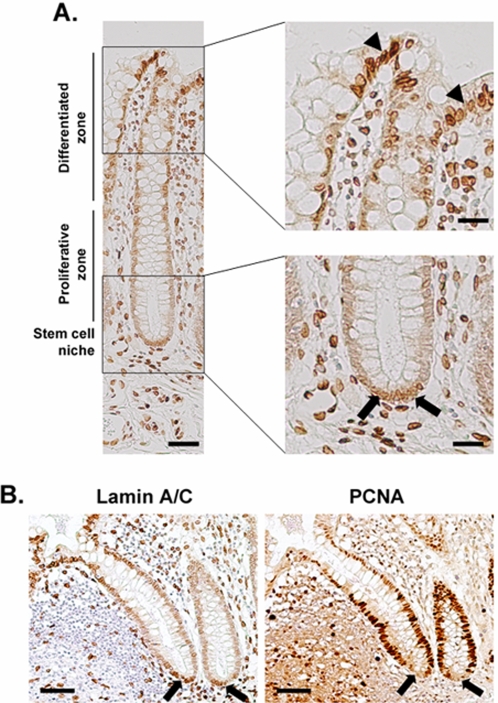
Lamin A/C is a biomarker of colonic stem cells. (A) Thin sections of formalin fixed, wax embedded samples of normal colon were stained following heat mediated antigen retrieval with a monoclonal antibody JoL2 against lamins A/C. Sections were lightly counterstained with Mayers Haemalum. Arrowheads indicate differentiated epithelium cells while arrows indicate the anticipated location of the stem cell niche in the base of the crypt. Scale bars = 150 µm (left-hand panel) & 50 µm (right-hand panels). (B) Serial sections of normal colon were stained with antibodies to either lamins A/C (JoL2) or PCNA (PC10) and processed as described above. Arrows indicate slowly dividing cells at the base of the crypts, which stain positively with JoL2 but negatively with PC10. Scale bars = 150 µm.

Before investigating whether A-type lamins influence tumour progression, we assessed the distribution of A-type lamins in normal colonic mucosa by staining tissue sections with a monoclonal antibody specific for these proteins. As expected [26], A-type lamins were highly expressed in the functionally differentiated epithelial layers and were also strongly expressed in surrounding stromal tissue and underlying muscle. Conversely, A-type lamin expression was very weak or absent from the majority of cells within the colonic crypts. However, A-type lamin expression was high in approximately five to ten cells at the base of the crypts (Figure 1A, arrows). The positively stained cells in the crypts were very similar in number and occupied a position corresponding to that generally understood to be the colonic epithelial stem cell niche [27], [28]. To further investigate the identity of these A-type lamin positive cells, serial sections were stained with antibodies against lamins A & C or the proliferation biomarker PCNA. The cells in the crypts that stained positively for PCNA were negative for A-type lamins, whilst the cells that were negative for PCNA were positive for A-type lamins (Figure 1B) implying that the small number of A-type lamin positive basal crypt cells could indeed be postulated to be stem cells. We also found that whilst antibodies that specifically detected lamin A did stain the proposed stem cell niche (but not cells in the adjacent transit amplifying zone), lamin C was not detected in either the stem cell niche or the transit amplifying cells, but was expressed in differentiated epithelial cells and underlying muscularis (Figure S1). Thus expression of lamin A in the absence of lamin C appears to define a group of cells in the basal region of the colonic crypt, corresponding to an area postulated to be the stem cell niche.

### Expression of A-type lamins is a hazard indicator in CRC

The Netherlands Cohort Study on Diet and Cancer [29] is a prospective cohort study. Initially, tissue samples from 819 patients were requested from 54 pathology laboratories throughout the Netherlands. Incident cases included those patients developing colorectal cancer between 1989 and 1993. Tumour material was not available for 5% of cases and of the remaining 775 available tissue samples, 737 contained sufficient tumour material for immunohistochemical analysis. Using standard parameters to test at the 5% significance level and with a 90% power, the minimum population size required to confidently detect small hazard ratios would be 516, thus it was safe to assume that the sample size was large enough to produce statistical significance.

**Figure 2:**
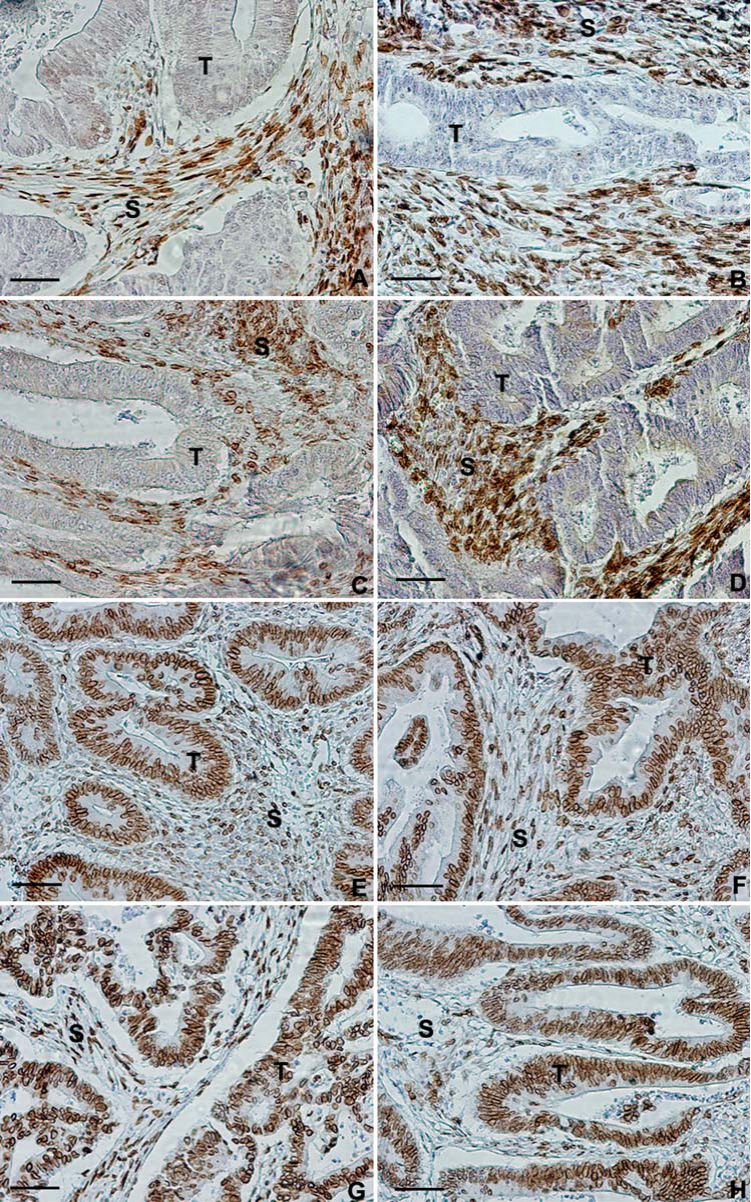
Differential expression of lamins A/C in colorectal cancer. 4 µm formalin-fixed, paraffin-embedded sections from 656 independent human colorectal adenocarcinomas were taken from patients participating in the Netherlands Cohort Study on Diet and Cancer. Tissue sections were subjected to immunohistochemistry using the JoL2 mouse monoclonal anti-human lamin A/C antibody before chromogen visualisation and light counterstaining with Mayers Haemalum to differentiate nuclei. A–D show positively staining stromal (S) tissue surrounding negatively staining tumour (T) tissue. (A) Moderately well differentiated stage I adenocarcinoma; (B) Moderately well differentiated stage II adenocarcinoma; (C) Moderately well differentiated stage III adenocarcinoma; (D) Moderately well differentiated stage IV adenocarcinoma. E–H show positively staining stromal (S) tissue surrounding positively staining tumour (T) tissue. (E) Moderately well differentiated stage I adenocarcinoma; (F) Moderately well differentiated stage II adenocarcinoma; (G) Moderately well differentiated stage III adenocarcinoma; (H) Moderately well differentiated stage IV adenocarcinoma. Scale bars = 30 µm.

Immunohistochemical staining to detect expression of lamins A/C following antigen retrieval was performed on all available samples. In all sections the stromal tissue surrounding the cancer was always strongly positive for lamins A/C providing an internal positive control. Using staining of stromal tissue as a criterion for successful antigen retrieval, 673 (91%) of the samples were available for scoring. We found that whilst there was some variation in the intensity of overall staining, nuclear lamin A/C expression within the tumour could be easily classified as either present (Figure 2E–H) or absent (Figure 2A–D) from the nucleus. It was also noted that in a small number of cases, there was sporadic cytoplasmic staining and these samples were scored as negative when nuclear staining was absent. Following blind scoring of slides, retrieval of patient and survival information from the database led the final number of unique cases to be reduced to 658 due to duplication of patient administration codes, and a further 2 cases were excluded upon being classified as signet ring tumours.

**Figure 3:**
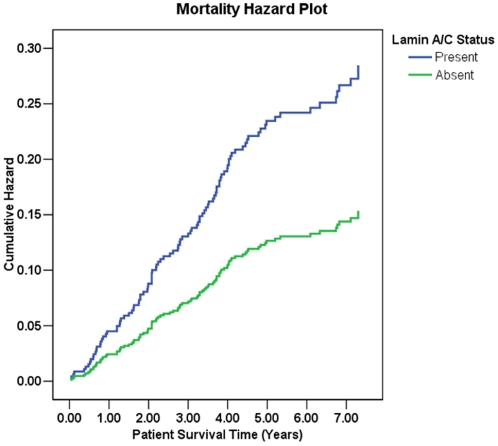
Mortality Hazard Plot for lamin A/C expression. Overall cumulative hazard analysis for presence of lamin A/C expression and colorectal cancer related mortality for stage I–III patients in the Netherlands Cohort Study on Diet and Cancer. Relative hazard ratio (HR) = 1.85 (95% C.I. = 1.16–2.97), *p* = 0.005 (Adjusted for gender and age at diagnosis).

During the follow-up period, 246 patients died, with 163 of these patients dying as a result of CRC. Of the 656 available specimens immunohistochemically assessed, nuclear lamin A/C expression was found to be positive in 463 (70%) patients and negative in 193 (30%) patients. Within the sample group of the patients who died of CRC related causes within the study period (ten years), 127 scored positive for lamins A/C, while 36 scored negative. Using Cox Proportional Hazard calculations [30], we found that patients expressing lamins A/C within the tumour were almost twice as likely to die (Hazard ratio [HR] 1.85; 95% confidence interval [C.I.] 1.16–2.97) of CRC related causes compared to clinicopathogically identical patients that were negative for lamins A/C (*p* = 0.005) (Figure 3). Thus expression of lamin A/C in a tumour was strongly correlated with CRC related death.

### Expression of lamin A in CRC cell lines promotes increased invasiveness

**Figure 4:**
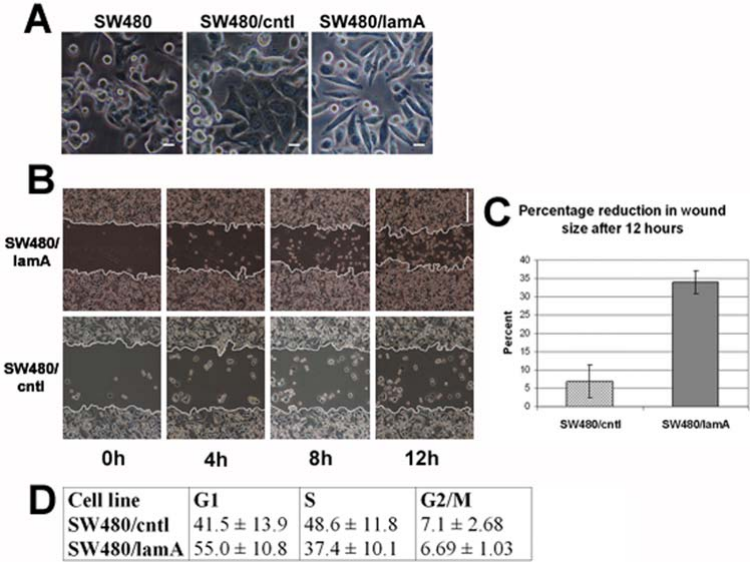
Expression of lamin A causes increased cell motility in SW480 colon cancer cells. (A) The morphology of untransfected SW480, SW480/cntl and SW480/lamA was compared by phase contrast microscopy. SW480/cntl and untransfected cells displayed a flattened morphology and multi-layered growth. By contrast, ectopic expression of GFP-lamin A (SW480/lamA) appeared to induce morphological changes, including the appearance of a spindle-like shape and growth as a monolayer. Scale bar = 10 µm. (B) Scratch wounds were made in 100% confluent cultures of SW480/cntl or SW480/lamA cells. Phase contrast images were taken every two hours over a 12 hour period from identical regions. Scale bar = 200 µm. (C) The wound size relative to the starting wound size was measured after 12 hours in three independent experiments and expressed as a percentage reduction in wound size+/−standard deviation (s.d.). Cell migration was ∼seven times faster in SW480/lamA compared to SW480/cntl cultures, p<0.005. (D) Cell cycle characteristics were investigated using flow cytometry. The proportion of cells in each phase of the cell cycle is given as mean±s.d. of three replicates. No appreciable differences in cells cycle dynamics between the two cells lines were detected.

The finding that expression of lamin A/C in patient tumours is closely correlated with CRC related mortality was unexpected and to some extent counter intuitive. Therefore, to understand why expression of lamins A/C increases the risk of death in CRC, we obtained a number of CRC cell lines and initially assessed them for lamin A/C status by immunoblotting. In one pre-metastatic cell line, SW480, lamin A was almost undetectable (Figure S2A, B) whilst levels of expression of lamins B_1_, B_2_ and C were similar to other CRC cell lines (e.g. HT29). SW480 was therefore selected for investigation. To determine how expression of lamin A might affect SW480, cultures were stably transfected with either GFP or a GFP-lamin A construct. Following stable transfection with GFP-lamin A moderate amounts of both endogenous lamin A as well as the fusion protein were detected, whereas in GFP transfected cultures, lamin A remained absent (Figure S2C, D). The morphology of cells in GFP and GFP-lamin A transfected cultures was also different. The morphology of cells in GFP transfected cultures (SW480/cntl) was indistinguishable from untransfected cultures (SW480), with many cells that were highly flattened or growing on top of each other. In contrast, in GFP-lamin A transfected cultures (SW480/lamA), cells had a more spindle like appearance and grew as a monolayer at low cell density (Figure 4A).

Migratory/metastatic behaviour in cancer cells is typically associated with phenotypic changes [31]. To investigate whether altered cell morphology correlated with altered migratory behaviour we performed cell motility assays on the cultures. Following scratch wounding, wound closure was seven times faster in cultures transfected with GFP-lamin A compared to cultures transfected with GFP (Figure 4B & C) or untransfected cells (not shown), showing that cells transfected with GFP-lamin A were indeed more motile than either cells transfected with GFP or untransfected cells. We investigated other features of their cellular behaviour which might alter as a result of expression of lamin A, including cell growth and division. Using flow cytometry we found no appreciable differences in cell cycle dynamics in GFP-lamin A versus GFP transfected cells (Figure 4D). Thus of the characteristics we investigated only cell morphology and motility were altered in the cells expressing lamin A.

In a recent study [32], expression of the actin-bundling protein, L-plastin in SW480 cells led to down-regulation of E-cadherin and increased invasiveness. Plastins are a family of actin-bundling proteins which are involved in organising the actin cytoskeleton and are expressed in a wide range of tissues [33], [34]. Enhanced expression of another plastin family member, T-plastin has been associated with both drug and radiation resistant cells. Of particular note is the significantly increased expression of T-plastin reported in cisplatin-resistant human cancers [34]. It follows that drug-resistant tumours are likely to be more aggressive. Our finding that the mortality rate in patients with lamin A/C expressing tumours is twice that of patients with lamin A/C negative tumours suggests that lamin A/C expression is also associated with more aggressive tumour behaviour. In addition, lamin A/C is thought to influence actin cytoskeletal organisation via interactions with linker proteins such as nesprins [4]. Furthermore, T-plastin activity has been reported in HT29 colon carcinoma cells [33] which are known to be lamin A positive (this paper). We questioned therefore whether expression of GFP-lamin A in SW480 cells caused downstream changes in T-plastin expression and whether T-plastin might be implicated in the increased invasiveness observed here.

**Figure 5:**
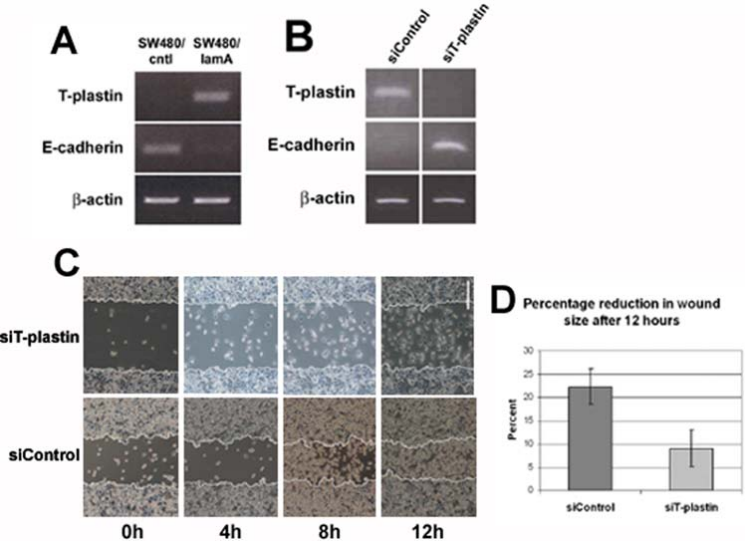
Expression of lamin A causes changes in expression of T-plastin and E-cadherin. (A) The expression of T-plastin and E-cadherin transcripts in SW480/cntl and SW480/lamA cells was investigated using semi-quantitative RT-PCR. Equal loading of starting material was determined by amplifying β-actin. SW480/lamA cells were found to express significantly higher levels of T-plastin mRNA compared to SW480/cntl, but significantly lower levels of E-cadherin mRNA compared to control cells (*p*<0.005). (B) SW480/lamA cultures were treated with scrambled siRNA or siRNA specific to T-plastin. 108h after treatment RNA was extracted and amplified by RT-PCR using primers specific to T-plastin and E-cadherin. One hundred percent knockdown of T-plastin was accompanied by re-expression of E-cadherin transcripts. (C, D) Alternatively, scratch wounding was performed on confluent cultures 108h after siRNA treatment and wound closure was measured as described in Figure 4B, C. Cell migration was >2 fold faster in cultures treated with scrambled siRNA compared to cultures treated with siT-plastin (*p*<0.05). Scale bar = 200 µm.

Changes in T-plastin and E-cadherin expression levels were investigated by RT-PCR. We found that T-plastin was not expressed in cells transfected with GFP but was present at high levels in cells expressing GFP-lamin A. In contrast, E-cadherin was expressed at high levels in cells expressing GFP alone but was absent from cells expressing GFP-lamin A (Figure 5A). Thus the increased motility of cells expressing GFP-lamin A likely arises because these cells are less adherent as a result of loss of expression of E-cadherin and exhibit enhanced actin dynamics. To determine whether the lamin A dependent up-regulated expression of T-plastin observed did indeed cause down-regulation of E-cadherin and increased cell motility, we performed siRNA knockdown of T-plastin in GFP-lamin A expressing SW480 cells. In cells treated with specific siRNA, T-plastin was undetectable and E-cadherin was up-regulated (Figure 5B). Importantly, following scratch wounding, wound closure was also significantly slower in cultures treated with T-plastin-specific siRNA compared to cultures treated with scrambled siRNA (Figure 5C, D). Thus up-regulated expression of T-plastin, arising from expression of lamin A affects E-cadherin expression and directly results in the more invasive properties of the lamin A transfected CRC cell line.

## Discussion

In this paper we report two novel and unexpected findings. Firstly, lamin A but not lamin C is a potential biomarker of the stem cell niche in the colonic crypt and secondly, expression of lamin A/C in CRC tissues is strongly correlated with CRC related mortality and therefore lamin A/C represents a novel and important prognostic biomarker in CRC.

The location of the stem cell niche in the colon has been extrapolated from cell kinetic and radiolabelling studies. By radiolabelling S-phase crypt cells with tritiated thymidine, the average migration velocity of cells in relation to their cell position has been measured. Using this approach, the origin of migration and, by extrapolation, the stem cell niche has been predicted to reside at the base of the crypt [35]. The number of stem cells occupying this niche approximates to between five to ten cells [36]. More recently, expression of the Wnt response element Lgr5 has been shown to define colonic stem cells and has been used to confirm that the stem cells occupy a niche at the base of the crypts [27]. Cells of an equivalent number and occupying precisely that niche are readily stained with antibodies detecting lamins A/C or lamin A but are not stained with antibodies specifically detecting lamin C. In contrast, in cells occupying the transit amplifying zone, expression of both lamin A and lamin C is absent. As expected [26], [37], [38], both lamins are readily detected in functional differentiated epithelium at the colonic mucosa. Consistent with the idea that lamin A is a potential biomarker of colonic stem cells, the cells at the base of the crypt which are positive for lamin A, are generally negative for the DNA replication protein PCNA, as would be expected in cells that are mostly quiescent [39]. That lamin A is a potential biomarker of adult stem cells has important general implications for disease. Lamins A/C are mutated in a wide range of degenerative diseases that are linked to premature ageing [3] and it has been suggested that one cause of degeneration in these diseases is loss of adult stem cell function [40]. That lamin A expression is noted in the adult stem cell niche of the colon, lends direct support to this hypothesis. The finding that lamin A may be a putative stem cell biomarker has further implications for our discovery that expression of lamin A/C in CRC is closely correlated with an increased risk of CRC-related mortality, since it could be considered feasible that cancer cells expressing lamin A may have a more stem cell-like phenotype and therefore be inherently more dangerous [41].

Our finding that expression of A-type lamins in CRC tissue is correlated with a two fold increase in CRC related mortality, compared to absence of A-type lamins, for the first time directly links these proteins to progression of a common disease. Previous studies have described altered expression of A-type lamins in a range of cancers, including cancers of the skin, lung, lymphatics and soft tissue [24], [37], [42]–[44]. However, none of these studies was able to link either absence or presence of A-type lamins to tumour progression, although at least one study [24] suggested that loss of expression of lamins A/C was correlated with enhanced proliferation rates in tumours. The other problem with these previous studies is that a limited number of samples were available for analysis and consequently study sizes fell below the threshold for reliable statistical analysis. In contrast, our current finding is based on a sufficiently large enough cohort to offer complete confidence in the significance levels reported.

Our finding is to some extent counter intuitive, in that expression of A-type lamins in various cells generally slows cell proliferation [21] while absence of A-type lamins can be correlated with a failure to undergo growth arrest at confluence [22]. Therefore, the null hypothesis used at the beginning of the study was that absence of A-type lamins would be correlated with increased risk of cancer-related death. Using model CRC cells lines we could not detect appreciable changes in the rate of cell proliferation in cells expressing lamin A compared to cells of an identical genetic background that lacked expression of lamin. However, we did detect significantly increased invasive properties when we compared lamin A expressing cells to cells in which expression of lamin A is absent. The invasive properties of these cells arises because the presence of lamin A causes a downstream up-regulation of T-plastin, which in turn leads to down-regulation of E-cadherin. Plastins are a recently described family of actin-bundling proteins which have been implicated in invasion/metastasis [34]. In the same cell line as used in this study, expression of L-plastin has been reported to lead to down-regulated expression of E-cadherin and increased invasiveness and has been closely correlated with metastasis [32]. Our current findings are entirely consistent with the previous study, but suggest, firstly that lamin A controls this pathway and secondly, that invasiveness can be induced by expression of T-plastin through the same mechanism. Interestingly, we also observed that endogenous lamin A was detected in SW480 cells upon stable expression of GFP-lamin A, whereas in the presence of GFP alone lamin A remained absent. It is well known that A-type lamins are susceptible to cleavage in the non-helical linker 2 region [3]. Lamin A exists in cells as either parallel dimmers or anti-parallel tetramers in which the N-terminal head domain overlaps the linker 2 region [45]. Since the GFP moiety is located at the N-terminus, perhaps this large protein protects linker 2 from proteolytic degradation and therefore stabilises both GFP-lamin A and endogenous lamin A. The corollary of this hypothesis is that absence of lamin A in SW480 cell lines is due to post-translational modification of the protein.

How the presence of lamin A influences the expression of an actin-bundling protein is the subject of downstream studies. However, two distinct mechanisms can be envisaged. It has recently been shown that A-type lamins directly influence the organisation of the cytoskeleton, the tensile strength of cells [46] and their ability to respond to stress [10]. This is achieved via the LINC complex, which mediates associations between the nuclear lamina and the cytoskeleton via the nesprins [4]. Therefore, one possibility is that the presence or absence of lamin A in a cancer cell might lead directly to cytoskeletal reorganisation via a feedback loop, which senses the tensile strength of the cell. Alternatively, lamin A has been shown to interact directly with a range of transcription factors [17] and its presence may directly influence the expression of T-plastin. Either way, the phenotypic outcome of this reorganisation is increased invasiveness and presumably increased metastatic potential.

In conclusion, we propose that the high risk of mortality from CRC related causes in patients who exhibit A-type lamin expression within their tumour arises because lamin A is an up-stream regulator of a pathway linking actin dynamics to loss of cell adhesion, thus leading to increased cell motility and consequently increased invasive potential of the tumour. This phenotype may in turn be reflective of a more stem cell-like property of the cancers. Whilst mutations in lamins A/C have been implicated in a wide range of rare genetic diseases [3], our current findings for the first time link expression of these proteins with progression of one of the commonest causes of cancer-related death in the Western world.

## Materials and Methods

### Immunohistochemistry

Paraffin sections (4 µm) of normal colonic mucosa and colorectal adenocarcinoma were de-paraffinised and rehydrated in xylene and ethanol. For antigen retrieval, sections were immersed in 3% H_2_O_2_ to quench endogenous peroxidase activity before being incubated in 0.01 M citrate buffer (pH 6.0) for 20 min at 90°C. Sections were then blocked with 5% normal goat serum followed by incubation with either JoL2, anti-lamin A/C mouse mAb [47] [1∶10], PC10, anti-PCNA mouse mAb [1∶100], RalC, anti-lamin C rabbit polyclonal antibody [24] [1∶100] or 133A2, anti-lamin A mouse mAb [48] [1∶100] overnight at 4°C. Sections were then incubated with biotinylated goat anti-mouse IgG diluted at 1∶400 for 45 min at room temperature. The standard ABC process was then performed according to instructions from Dako (DakoCytomation, Glostrup, Denmark). Diaminobenzidine was used as a chromogen followed by light counterstaining with Mayers Haemalum.

### Cell culture

The human pre-metastatic colon adenocarcinoma cell lines HT29 and SW480 were obtained from the European Collection of Cell Cultures, UK. HT29 were cultured in McCoy's 5A medium (Sigma, UK) supplemented with 10% FBS, 2 mM L-Glutamine, 100 U/ml penicillin and 100 µg/ml streptomycin and maintained in a humidified environment at 37°C with 5% CO_2_. SW480 cells and transfected derivatives were grown in Leibovitz-15 (L-15) medium (Invitrogen, UK) supplemented with 10% FBS, 100 U/ml penicillin and 100 µg/ml streptomycin and maintained in a humidified environment at 37°C without CO_2_.

### Stable transfection of GFP-reporters into SW480 colon adenocarcinoma cells

SW480 cells were transfected with DNA constructs encoding a fusion protein of EGFP-lamin A full-length (gift from Dr M. Izumi, Institute of Physical and Chemical Research, Saitama, Japan) and EGFP. Cells were grown in 6-well plates until 60% confluent and transfected with either 3 µg EGFP-lamin A or 1 µg EGFP using GeneJammer® transfection reagent (Stratagene, La Jolla, CA) according to the manufacturer's instructions. Five days post-transfection, cells were split 1∶3. Geneticin® (G-418 sulphate, Invitrogen) selective antibiotic was added to a final concentration of 200 µg/ml 24h later. Fresh antibiotic was added every 72h when the media was changed. The selection of transfected colonies began when all cells from the negative control (DNA construct replaced by 1 µl ddH_2_0 in transfection mixture) died off. Surviving transfectants were cloned out by limited dilution in 96-well plates and scaled up under constant antibiotic selection. Stably transfected clones were identified by screening four weeks after antibiotic selection was removed. All subsequent experiments were done in the absence of selective antibiotic. Cellular characterisation was completed on a particular EGFP-lamin A clone, known as SW480/lamA and a particular EGFP clone, termed SW480/cntl which were selected based on their moderate EGFP-reporter expression, ascertained by western blot. In addition the motility of transfected cells (as determined by scratch wound assay) was confirmed using a further three EGFP-lamin A clones and a second EGFP clone.

### Indirect immunofluorescence & confocal microscopy

HT29 & SW480 cells grown to 70% confluency on glass coverslips pre-coated with poly-D-lysine (0.01 mg/ml) were fixed with ice-cold methanol/acetone (1∶1, v/v) for 10 min at 4°C. Lamin A mAb, JoL4 [47] was applied undiluted for 1h. Incubation with FITC-conjugated donkey anti-mouse IgG secondary antibodies (Jackson ImmunoResearch, PA) diluted 1∶50 in 1% NCS/PBS was for 1h. Coverslips were mounted in Mowiol media containing 2.5% 1,4-diazabicyclo[2.2.2]octane (DABCO)/1 µg/ml DAPI. Images were captured using a Zeiss Axioskop microscope equipped with a Plan-Neofluar 40×/1.3 oil immersion lens and fitted with a Bio-Rad Radiance 2000 confocal scanning system, operated by LaserSharp 2000™ software (Carl Zeiss). Z-series were collected in Sequential mode using Kalman averaging (4 times) at a resolution of 1024×1024 pixels with an additional 2× digital zoom. Images were projected into z-stacks.

SW480/lamA and SW480/cntl cells were similarly grown to 70% confluency, but fixed with pre-warmed (37°C) 4% formaldehyde in PBS for 12 min, permeabilised with 0.5% Triton X-100 in PBS for 5 min and blocked in 1% newborn calf serum (NCS) in PBS for 30 min. GFP-reporter expression was viewed using a Zeiss LSM 510-META microscope equipped with a Plan-Neofluar 40×/1.3 oil immersion lens. Images were collected with a Zeiss Axiocam CCD camera directed by Zeiss Axiovision software, version 3.0. All images were organised using Adobe® Photoshop® 7.0 (Adobe Systems, CA).

### One-dimensional SDS-PAGE and immunoblotting

Whole cell extracts were made from cultured cells harvested at 80% confluency. Cells were washed with 2× PBS and scraped from the culture surface. Pellets were re-suspended in 500 µl Lysis buffer per 7×10^6^ cells [10 mM Tris-HCl (pH 7.4), 10 mM KCl, 3 mM MgCl_2_, 0.1% Triton X-100 and 1× Protease inhibitor cocktail (Sigma)] and then incubated with 0.1 units/µl DNase I on ice for 10 min. Cell lysates were dissolved in 500 µl 2× Sample buffer [125 mM Tris-HCl (pH 6.8), 2% SDS, 2 mM DTT, 20% Glycerol, 5% β-mercaptoethanol and 0.25% Bromophenol blue (w/v)], boiled at 95°C for 3 min and centrifuged at 14,000×g for 3 min. Proteins were resolved on 10% SDS-PAGE gels according to Laemmli, 1970 [49] and electrophoretically transferred onto nitrocellulose membranes (Protran®, Schleider and Schuell, NH). Membranes were blocked with 4% skimmed milk powder (w/v) in blot rinse buffer for 16h at 4°C with constant agitation and immunoblotted with anti-lamin A mAb, JoL4 [1∶200]; rabbit polyclonal antibody RaLC [1∶150]; goat polyclonal anti-lamin B1 (Santa Cruz Biotechnology, CA) [1∶250] and mouse monoclonal anti-lamin B2, LN43 [50] [1∶250]. β-actin mAb, clone AC-40 (Sigma) [1∶1750] was used as a control for loading. Secondary antibodies were HRP-conjugated donkey anti-mouse, donkey anti-rabbit or donkey anti-goat IgG used at a concentration of 1∶2000. Nitrocellulose membranes were exposed to ECL™ western blotting reagents (GE Healthcare, UK) and immunoreactivity was measured by recovering the signal of chemiluminescence on Hyperfilm™ ECL films (GE Healthcare) using a Compact X4 Automatic X-ray Film Processor (Xograph Imaging Systems Ltd, UK). Differences in signal were quantified by densitometry using Image Gauge version 4.0 (Fujifilm).

### Scratch wound assay

Scratch wounds more than 5mm in length and of equal thickness were made in 100% confluent cultures of SW480/cntl or SW480/lamA cells with a 10 µl disposable eppendorf tip. Phase contrast images were taken every two hours over a 12 hour period from identical regions. The wound size after 12h relative to the starting wound size was measured using Zeiss LSM Image Browser software, version 3.1, in three independent experiments. This experiment was repeated on an additional three EGFP-lamin A transfected SW480 clonal lines and a second EGFP-transfected SW480 clonal line (data not shown).

### Flow cytometry

Cells were processed at 70% confluency using the CycleTEST™ PLUS DNA Reagent kit (Becton Dickinson, NJ) according to the manufacturer's instructions. Cells were analysed on a Becton Dickinson FACSCalibur instrument and data was collected for 20,000 single cell events. The percentage of cells in G1, S and G2/M phases of the cell cycle was determined by the Dean/Jett/Fox model using FlowJo software (Treestar, OR).

### RNA isolation and semi-quantitative RT-PCR

Total RNA was isolated from 70% confluent cultures of SW480/lamA and SW480/cntl cells using Trizol (Invitrogen). Additional extractions were also made from SW480/lamA cells 108h post-transfection with scrambled or T-plastin siRNAs. cDNA was synthesized using Promega's Reverse Transcription System and Avian Myeblastosis Virus - Reverse Transcriptase (AMV-RT) [Promega, UK]. In control samples AMV-RT was replaced by an equivalent volume of nuclease-free water. The PCR amplifications were performed in triplicate in 25 µl reactions containing 1× PCR Master Mix (Promega), 0.4 pmol/µl sense primer, 0.4 pmol/µl antisense primer and 2 µl cDNA template. Equal loading of starting material was verified by monitoring the transcriptional activity of β-actin. PCR profiles for E-cadherin, T-plastin and β-actin were carried out on an Eppendorf Mastercycler® Gradient Thermal Cycler (Eppendorf, UK) as follows: 94°C for 2 minutes, 26 cycles at 94°C for 45 seconds, 55°C (E-cadherin) or 58°C (T-plastin) for 40 seconds or 60°C (β-actin) for 1 minute and 72°C for 1 minute, and finally 72°C for 5 minutes. Primer sequences were: T-plastin sense 5′-GCATCTTCCCTCTCATACCC-3′,

T-plastin antisense 5′-GCAAACAGCTTGACAAAGCA-3′,

E-cadherin sense 5′-CCAAGTGCCTGCTTTTGATG-3′,

E-cadherin antisense 5′-CACAATTATCAGCACCCACAC-3′,

β-actin sense 5′-GGCACCACACCTTCTACAATGAGC-3′ and

β-actin antisense 5′-CGTCATACTCCTGCTTGCTGATCCAC-3′.

Each product was sequenced with the corresponding antisense primer using an ABI Prism® 377 XL automated DNA sequencer (Applied Biosystems, CA) and verified using the Nucleotide-nucleotide BLAST database (BLASTN 2.2.11).

### siRNA transfections

SW480/lamA cells were seeded at a density of 2.5×10^5^ cells/flask in T-25 flasks 24h before transfection. Cells were washed and normal culture media was replaced with 3.6 ml antibiotic-free media 2h before transfection. Cells were treated with a transfection mixture containing 400 µl L-15 medium, 20 µl T-plastin siRNA (*Silencer®* Pre-designed siRNA ID#143988, Ambion) [20 µM] or scrambled siRNA (Ambion) [20 µM] and 20 µl Oligofectamine reagent (Invitrogen). Media was changed after 24h and replaced with normal culture medium (plus antibiotics). Transfection efficiency was maximal after 108h, as determined by semi-quantitative RT-PCR. The sequences of the T-plastin-specific siRNA duplex were: sense: 5′-CCACGGAUAGAUAUUAACAtt-3′ and antisense: 5′-UGUUAAUAUCUAUCCGUGGtt-3′.

### Statistical Analysis

Baseline characteristics of patients, tumours as well as tumour biology variables were compared by Student's t-test (continuous variables) and Chi-squared (χ^2^) tests (categorical variables) to the study variable (lamin A/C expression). Hazard ratios for disease and 95% confidence intervals were calculated by conditional logistic regression. In multivariate analyses, missing values were treated as a separate category or excluded (categorical variables) or given the median-value (continuous variables). All statistical tests and corresponding *p*-values reported were for two-sided tests and *p*-values of less than 0.05 were considered statistically significant. SPSS version 12.0 (Chicago, IL) was used for all statistical analyses.

### Consent and Approval

Specimen collection and archiving of patient data was performed using written informed consent and approved by the national ethical committee of The Netherlands.

## Supporting Information

Figure S1 Four micron serial sections of normal colonic epithelium were immunohistochemically stained for lamin A (A & C) and lamin C (B & D) using the 133A2 monoclonal and the RaLC polyclonal antibodies respectively. Arrowheads indicate functional differentiated cells and arrows indicate cells within the proposed stem cell niche. Scale bars = 50 µm.
(3.10 MB DOC)

Figure S2 Stable transfection of SW480 cells with GFP constructs. (A) A- and B- type lamin expression was compared in two colon adenocarcinoma cell lines - HT29 and SW480 - by either immunoblotting using antibodies JoL4 (anti-lamin A), RaLC (anti-lamin C), anti-lamin B1 and LN43 (anti-lamin B2) or (B) immunofluorescence using JoL4. Lamin A expression was almost undetectable in SW480 cells compared to HT29 cells. Consequently SW480 cells were selected for further investigation. There were no differences in the expression of other lamin isoforms between the two cell lines. (C) SW480 cells were transfected with DNA constructs encoding EGFP-lamin A full-length (SW480/lamA) or EGFP as a control (SW480/cntl). One hundred percent stable transfection was achieved for both constructs as a result of antibiotic selection. The level of total lamin A in each transfected culture was determined by immunoblotting using JoL2 (anti-lamin A/C). β-actin was a loading control. (D) Alternatively, the distribution of the fusion protein was investigated by fluorescence microscopy. Scale bars = 10 µm.
(0.68 MB TIF)
