# Supplementary figures and images for: Lamin A/C Is a Risk Biomarker in Colorectal Cancer
Source: PLoS One. 2008 Aug 20;3(8):e2988. doi: 10.1371/journal.pone.0002988 (PMC2496895; doi:10.1371/journal.pone.0002988)

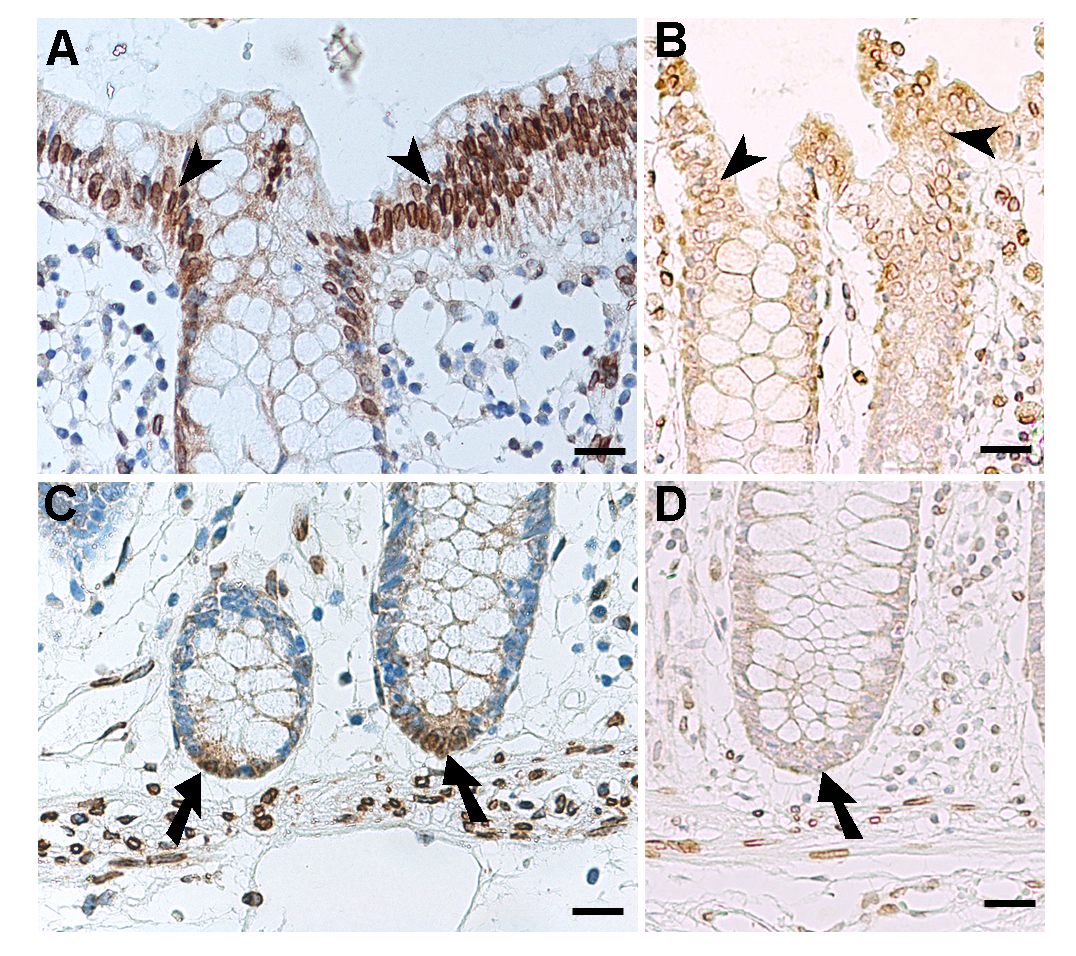

Supplement: Figure S1 — Four micron serial sections of normal colonic epithelium were immunohistochemically stained for lamin A (A & C) and lamin C (B & D) using the 133A2 monoclonal and the RaLC polyclonal antibodies respectively. Arrowheads indicate functional differentiated cells and arrows indicate cells within the proposed stem cell niche. Scale bars = 50 µm. (3.10 MB DOC) [file pone.0002988.s001.tif]

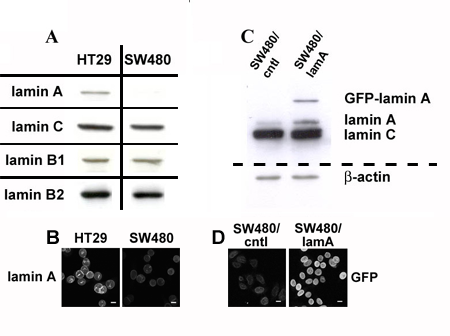

Supplement: Figure S2 — Stable transfection of SW480 cells with GFP constructs. (A) A- and B- type lamin expression was compared in two colon adenocarcinoma cell lines - HT29 and SW480 - by either immunoblotting using antibodies JoL4 (anti-lamin A), RaLC (anti-lamin C), anti-lamin B1 and LN43 (anti-lamin B2) or (B) immunofluorescence using JoL4. Lamin A expression was almost undetectable in SW480 cells compared to HT29 cells. Consequently SW480 cells were selected for further investigation. There were no differences in the expression of other lamin isoforms between the two cell lines. (C) SW480 cells were transfected with DNA constructs encoding EGFP-lamin A full-length (SW480/lamA) or EGFP as a control (SW480/cntl). One hundred percent stable transfection was achieved for both constructs as a result of antibiotic selection. The level of total lamin A in each transfected culture was determined by immunoblotting using JoL2 (anti-lamin A/C). β-actin was a loading control. (D) Alternatively, the distribution of the fusion protein was investigated by fluorescence microscopy. Scale bars = 10 µm. (0.68 MB TIF) [file pone.0002988.s002.tif]
